# Task aftereffect reorganization of resting state functional brain networks in healthy aging and mild cognitive impairment

**DOI:** 10.3389/fnagi.2022.1061254

**Published:** 2023-01-11

**Authors:** Rok Požar, Katherine Kero, Tim Martin, Bruno Giordani, Voyko Kavcic

**Affiliations:** ^1^Faculty of Mathematics, Natural Sciences and Information Technologies, University of Primorska, Koper, Slovenia; ^2^Andrej Marušič Institute, University of Primorska, Koper, Slovenia; ^3^Institute of Mathematics, Physics and Mechanics, Ljubljana, Slovenia; ^4^Institute of Gerontology, Wayne State University, Detroit, MI, United States; ^5^Department of Psychological Science, Kennesaw State University, Kennesaw, GA, United States; ^6^Michigan Alzheimer’s Disease Research Center, University of Michigan, Ann Arbor, MI, United States; ^7^International Institute of Applied Gerontology, Ljubljana, Slovenia

**Keywords:** resting-state electroencephalography, mild cognitive impairment, brain network, task aftereffect model, neuropsychology

## Abstract

The view of the human brain as a complex network has led to considerable advances in understanding the brain’s network organization during rest and task, in both health and disease. Here, we propose that examining brain networks within the task aftereffect model, in which we compare resting-state networks immediately before and after a cognitive engagement task, may enhance differentiation between those with normal cognition and those with increased risk for cognitive decline. We validated this model by comparing the pre- and post-task resting-state functional network organization of neurologically intact elderly and those with mild cognitive impairment (MCI) derived from electroencephalography recordings. We have demonstrated that a cognitive task among MCI patients induced, compared to healthy controls, a significantly higher increment in global network integration with an increased number of vertices taking a more central role within the network from the pre- to post-task resting state. Such modified network organization may aid cognitive performance by increasing the flow of information through the most central vertices among MCI patients who seem to require more communication and recruitment across brain areas to maintain or improve task performance. This could indicate that MCI patients are engaged in compensatory activation, especially as both groups did not differ in their task performance. In addition, no significant group differences were observed in network topology during the pre-task resting state. Our findings thus emphasize that the task aftereffect model is relevant for enhancing the identification of network topology abnormalities related to cognitive decline, and also for improving our understanding of inherent differences in brain network organization for MCI patients, and could therefore represent a valid marker of cortical capacity and/or cortical health.

## Introduction

The human brain, as perhaps the most complex body system, is in a constant flux of transitions. Even without external stimulation, the integration of information between specialized, functionally connected brain areas continues. Graph theory provides a simple framework to model a complex system as a network, consisting of vertices and edges that represent the basic building blocks of the system and their relations. The view of the human brain as a network has led to considerable advances in understanding the topological organization of the human brain during rest and task, in both health and disease (Bullmore and Sporns, 2009; Stam and Van Straaten, 2012; Stam, 2014). There is strong evidence that successful information flow within the network, ensuring proper cognitive functioning, is supported by an optimal balance between local processing and global integration (Bullmore and Sporns, 2012; Bassett and Bullmore, 2016). In this regard, vertices with a more central role in the network have a special significance in controlling the information flow process and thus in global network communication (van den Heuvel and Sporns, 2011, 2013).

Mild cognitive impairment (MCI) is viewed as an early transitional state between normal aging and the appearance of dementia. This diagnosis is linked to reasonable ability to maintain independent activities of daily living, but with early evidence of memory loss or other cognitive abilities (e.g., language, executive functioning, and visual/spatial ability). Investigating functional brain networks in patients with MCI, using resting-state electroencephalography (EEG) recordings, seems a promising approach and one that might provide a reasonable biological marker for differentiation between healthy controls and persons with MCI. Several previous EEG studies have reported that the optimal network organization is disrupted in MCI with a deviation to a less integrated topology (Xu et al., 2014; Zeng et al., 2015; Požar et al., 2020). These results have been primarily obtained from a single resting-state EEG recording without previous stimulation.

Here, we examine brain network organization within the *task aftereffect model*, in which we compare resting-state brain network organization immediately before and after a cognitive engagement task. There is some evidence that this model may enhance differentiation between those with normal cognition and those with MCI. Although baseline, resting-state EEG is considered to represent intrinsic neural activity that is highly stable, our research group was the first to show that, compared to healthy controls, older adults with MCI have a significantly higher reduction in spectral power from the pre- to post-task resting-state in specific cortical areas important for memory and problem solving (Kavcic et al., 2021). This finding indicates that the return to the baseline resting-state EEG is time-consuming and may index the efficacy of cognitive processing and/or cognitive health. Subsequently, only one other study has examined the interplay between brain network organization in MCI identified with resting-state EEG data before and after a cognitive task (Youssef et al., 2021). That study revealed a global reorganization from the pre- to post-task resting-state of the patients’ brain networks when compared to healthy controls. Thus, the task aftereffect model could reveal new inherent differences in brain organization for patients with MCI. However, it is not clearly understood whether this reorganization progressed towards increased or decreased network integration in MCI participants (see Discussion). Another issue that has not been addressed is how the distribution of the most crucial vertices with a more central role in the brain network may change in MCI under the task aftereffect model.

The aim of the present study was therefore to investigate if, and in which direction, the organization of functional connectivity edges changes within the network from the pre- to post-task resting-state, and to consider whether the presence of MCI modulates these functional connectivity patterns. To this end, we compared the pre- and post-task resting-state EEG functional network organization of neurologically intact elderly and those with MCI using conventional approach based on weighted graphs, as well as maximum spanning tree approach.

We computed several network measures to assess the degree of local and global network integration, as well as relative importance of vertices. We hypothesized that brain networks would be reorganized between the pre- and post-task resting states among elderly persons, primarily driven by patients with MCI who would demonstrate a variation in distribution of vertices with a more central role within the network.

## Materials and methods

### Participants

In this study, we explored a dataset that was previously collected in a study of 99 African Americans (Kavcic et al., 2021). Participants were recruited out of the volunteer pool of Healthier Black Elders Center, a joint collaboration between Wayne State University’s Institute of Gerontology and University of Michigan’s Institute of Social Research, and the Michigan Alzheimer’s Disease Research Center (MADRC). Participants were enrolled based on their responses to a question included in the health screening forms asking if they had experienced a change in memory or other cognitive areas over the past year, but not so severe as to interfere with their ability to complete daily activities. Of 99 participants 41 were determined to meet MCI criteria, based on a MADRC consensus conference meeting criteria established by the National Alzheimer’s Disease Coordinating Center and using measures from the Unified Dataset (Beekly et al., 2007). All participants were consented and procedures were approved by the Wayne State University Research Subjects Review Board and the University of Michigan Medical School Review Board (see Table 1 for demographics of the participants).

**Table 1:** Demographic characteristics and discrimination task of control and MCI subject groups.

	Controls (*N* = 58)		MCI (*N* = 41)		*p* Value^*^
	Mean	SD	Mean	SD	
Age (years)	71.10	6.18	73.73	7.19	0.0516
Education (years)	15.26	2.34	14.46	2.42	0.0897
Gender (% female)	91%	–	85%	–	–
MDDT (time)	0.75	0.14	0.75	0.13	0.3979

MDDT, motion direction discrimination task; SD, standard deviation.

* The *p* value was obtained using Mann–Whitney *U*-tests for independent samples.

### Electroencephalography recordings

Scalp electroencephalographic activity was recorded before and after a motion direction discrimination task for at least 3 min of resting-state with eyes closed using Brain Vision (Brain Vision, Inc.) equipment. We used the high-density Acti Cap (64 active electrodes) modified according to the International 10–20 System. The recording locations included eight midline sites, with the FCz electrode as an on-line reference and a ground at midline location AFz. Low and high pass filter settings were 0.1 and 70 Hz, respectively. The cutoff frequencies for these filters were set at 3 dB down; the roll-off was 12 dB per octave at both sides. Impedances were maintained below 10 kΩ for each channel and balanced across all channels within a 5 kΩ range. The sampling rate was 500 Hz with a 32-bit resolution.

### Motion direction discrimination task recordings

Global motion stimuli were presented on the computer monitor and consisted of white dots subtending 0.125^°^ of visual angle presented within a circular aperture 10^°^ in radius on a uniform black background. Dot motion was controlled by custom software, programmed in C language, compiled with the MinGW compiler,^1^ on a Windows XP computer. Motion duration was 500 ms, with either rightward or leftward direction on randomly ordered trials and button press direction responses collected on a laptop (see Table 1 for discrimination task results of the participants).

### Electroencephalography data analyses

To check the quality of the resting state eyes-closed EEG signal, the EEG data were inspected by using Brain Vision Analyzer 2.2. Initially, we applied an off-line raw data inspection procedure to identify and remove segments of EEG recording that were contaminated with excessive noise, saturation, or lack of EEG signal activity. Where needed, we applied independent component analyses to correct for eyeblinks and/or lateral eyes movements. The visually detected bad channels were interpolated using the spherical method. The EEG data were then segmented into consecutive epochs (1,024 data points) for further off-line analysis. The epochs were identified as acceptable by an automatic artifact rejection procedure, using a rejection criterion of 100 mV on any channel affected by artifacts (muscular, instrumental). For each subject 30 artifact-free epochs were used to compute functional connectivity in the following frequency bands: delta (0.5–4 Hz), theta (4–8 Hz), lower alpha (8–10 Hz), upper alpha (10–13 Hz) and beta (13–20 Hz). Functional connectivity between each pair of EEG channels was assessed by the phase lag index (PLI; Stam et al., 2007) for all epochs of each subject and stored in the corresponding 64×64 matrix. The PLI measures the asymmetry of the distribution of instantaneous phase differences between two EEG signals and reduces the influence of volume conduction and reference electrode.

### Graph analysis

Each functional connectivity matrix with PLI values represents a weighted brain graph in which each vertex represents one electrode and the functional connectivity edge between any two vertices represents the relation between the respective electrodes weighted by the strength of functional connectivity.

#### Traditional graph analysis

We computed two traditional network measures of a weighted brain graph to assess the degree of network organization: the weighted clustering coefficient and the weighted distance.^2^ Table 2 shows an overview of these measures. For subsequent analysis, we used mean weighted clustering coefficient over all vertices, called *global weighted clustering coefficient*, to identify the tendency of vertices to form local clusters and mean weighted distance over all pairs of distinct vertices, called *weighted characteristic path length*, to measure how easily information can be transferred across the network. In addition, each of these two measures was normalized by its mean over all frequency bands (Rossini et al., 2020).

**Table 2:** Traditional measures on a weighted brain graph.

Graph concept	Explanation
*Intensity*	The intensity of a triangle is the geometric mean of its normalized edge weights (each weight is normalized by the maximum weight in a graph).
*Weighted clustering coefficient*	The weighted clustering coefficient of a node in a weighted brain graph is the average intensity of triangles in which that node participates. It reflects the tendency to which edges tend to cluster into tightly connected neighborhoods.
*Inverse weighted length*	The inverse weighted length of a path in a weighted brain graph is the sum of the reciprocals of its edge weights.
*Weighted distance*	The weighted distance between two distinct vertices in a weighted (brain) graph is the shortest inverse weighted length of any path between them.

#### Maximum spanning tree analysis

The calculated PLI matrices served as input for Kruskal’s algorithm (Kruskal, 1956) to compute a maximum spanning tree. A *maximum spanning tree* (MST) of the brain graph is a (sub)graph consisting of all vertices of the original graph and a smallest subset of edges that connects all the vertices and maximizes the sum of edge-weights.^3^ A MST represents, by definition, the backbone of the brain network in the sense that it captures the strongest functional connectivity edges and ensures that all networks to be compared have the same number of vertices and the same number of edges (Tewarie et al., 2015). The obtained trees contained 64 vertices and 63 edges. The corresponding edge-weights were ignored in subsequent characterizations. In this way, we can guarantee that no differences between connection density or functional strength were present between participants.

**Figure 1:**
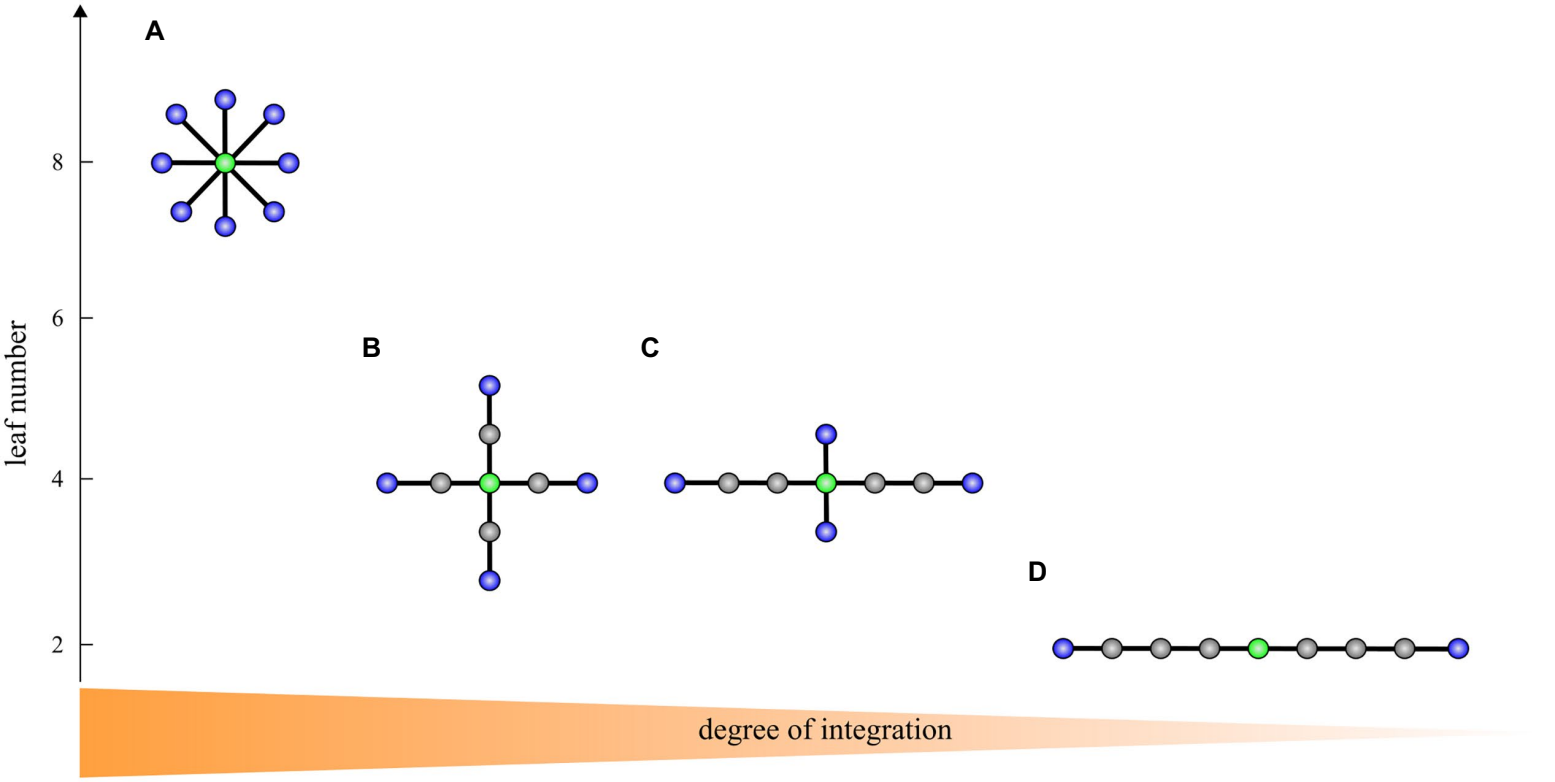
A transition from a star-like topology **(A)** to a path-like topology **(D)** with two intermediate topologies **(B,C)** corresponding to the brain network integration from highest to lowest on trees with nine vertices. Blue vertices: leaves. Green vertices: vertices with the highest betweenness centrality and lowest eccentricity representing the most critical/central vertices in the tree. In **A**, the leaf number is 8, the diameter is 2, the maximum betweenness centrality is 28, and the mean eccentricity is 1.9. In **B**, the leaf number is 4, the diameter is 4, the maximum betweenness centrality is 24, and the mean eccentricity is 3.3. In **C**, the leaf number is 4, the diameter is 6, the maximum betweenness centrality is 22, and the mean eccentricity is 4.6. In **D**, the leaf number is 2, the diameter is 8, the maximum betweenness centrality is 16, and the mean eccentricity is 6.2. The figure is adapted from van Lutterveld et al. (2017).

To characterize brain network topology, we computed the following MST measures indicating network integration and efficiency: the leaf number, diameter, eccentricity, and betweenness centrality. Table 3 shows an overview of these measures and different tree topologies with decreasing degree of integration are displayed in Figure 1. For subsequent analysis, each measure was normalized by its maximal possible value, yielding values ranging from 0 to 1. We also computed mean eccentricity over all vertices to identify the eccentricity of the whole tree. In addition, we used the maximum betweenness centrality over all vertices as global characteristics of a tree.

**Table 3:** Basic concepts and measures on a tree.

Graph concept	Explanation
*Leaf*	A vertex connected by an edge to precisely one other vertex.
*Leaf number*	The number of leaves. It provides information about network centralization.
*Distance*	The distance between two distinct vertices in a tree is the number of edges on the path between them (note that there is a unique path between any two vertices in a tree).
*Diameter*	The greatest distance between any two distinct vertices in the tree. It reflects the communication efficiency of global network topology.
*Eccentricity*	The eccentricity of a vertex in a tree is the greatest distance from that vertex to any other vertex in a tree. It measures relative importance of a vertex for global communication, and may indicate the critical vertices in a tree. The lower eccentricity value indicates that the vertex is more central since it is closer to the center of the tree.
*Betweenness centrality*	In a tree, the betweenness centrality of a vertex is the number of paths in the tree that include that vertex as an intermediate vertex. It measures relative importance of a vertex for global communication, and may indicate the critical vertices in a tree. A vertex with higher betweenness centrality exhibits higher domination over the information flows across the network. The maximum betweenness centrality over all vertices characterizes the importance of the most central vertex.

A large leaf number and small diameter characterizes a more star-like, centralized topology. A *star* consists of a central vertex and several leaves connected to this central vertex (Figure 1A). In contrast, a small number of leaves and large diameter reflects a more path-like, decentralized network topology. A *path* consists of a sequence of (different) vertices such that every two consecutive vertices are connected by an edge (Figure 1D). An optimal organization is probably somewhere between a path-like topology and star-like topology. A path is weakly integrated and is thus inefficient in the transfer of information. A star is highly integrated, and consequently, in such a graph information can be efficiently transferred, however, the central vertex whose betweenness centrality is maximal possible may experience an information overload. Overall, in a highly integrated network, leaf number and maximum betweenness centrality are large, while diameter and mean eccentricity are small. On the other hand, in a less integrated network, leaf number and maximum betweenness centrality are small, while diameter and eccentricity are large.

### Statistical analysis

All statistical analyses were performed in *Matlab v2016b*. First, network measures were averaged across the epochs per subject prior to statistical analysis. Then, outliers in network measures for each frequency band were detected and excluded from the subsequent analyses. To find outlier values, we used an iterative implementation of the Grubbs’s test (Grubbs, 1969). Accordingly, in the delta frequency band, one subject from each group was excluded for the leaf number, one subject from the control group for the maximum betweenness centrality and one subject from the MCI group for the mean weighted clustering coefficient. For the theta frequency band, one subject from each group was excluded for the mean weighted clustering coefficient and the mean weighted distance. For the lower alpha frequency band, two subjects from the control group were excluded for the mean weighted clustering coefficient. For the upper alpha frequency band, two subjects from the control group and one from the MCI group were excluded for the mean weighted clustering coefficient, and one subject from the control group for the mean weighted distance. For the beta frequency band, two subjects from the control group were excluded in the leaf number and three subjects from the control group in the maximum betweenness centrality. All results include the number of usable subjects for each graph measure.

To study network topology in the pre-task resting-state, permutation tests were applied to identify between-group differences in network measures at each of the five frequency bands (Nichols and Holmes, 2002). A correction for multiple comparisons across frequency bands was performed by the false discovery rate (FDR; Benjamini and Hochberg, 1995).

For each frequency band we employed linear mixed models (LMM) to describe the influence of the task and group in networks measures and also to quantify differences in the effect of the task between the two groups. LMM considers the within-subject variability by including a random intercept term associated with each subject (Laird and Ware, 1982). All LMM included the factor timepoint (the pre-task vs. the post-task), the factor group (the control vs. the MCI group), and the interaction between both factors. Age, gender and education were included as covariates. Each LMM also involved a random intercept term for the effect of the task for each subject. A correction for multiple comparisons across frequency bands was performed by the false discovery rate. The effects of the timepoints, group, or interaction were considered significant if the FDR-corrected p value was less than 0.05. If the effect of the timepoints/interaction was significant, we modeled control and MCI groups separately to evaluate the timepoint effect in each group.

To complement the global analysis, we explored significant differences between groups based on eccentricity at the vertex level. If the interaction effect in mean eccentricity was significant, we computed vertex eccentricity post-task/pre-task ratios to assess the changes in eccentricity from the pre- to post-task period. To quantify differences between groups on these vertex eccentricity ratios, we performed permutation tests based on maximum *t*-test statistic to control for family-wise-error (FWE) *p* values, i.e., correcting for multiple comparisons across vertices.

## Results

The demographic characteristics of participants and discrimination task are given in Table 1. The Motion direction discrimination task did not differ significantly between the groups.

### Graph analysis

Resting state network measures before and after cognitive engagement are presented in Supplementary Table 1 separately for normal controls and MCI.

#### Functional brain organization at the pre-task resting-state EEG

No significant group differences were observed for the network measures in any frequency band after FDR correction for number of frequency bands.

#### Functional brain reorganization from the pre- to post-task resting state EEG

**Figure 2:**
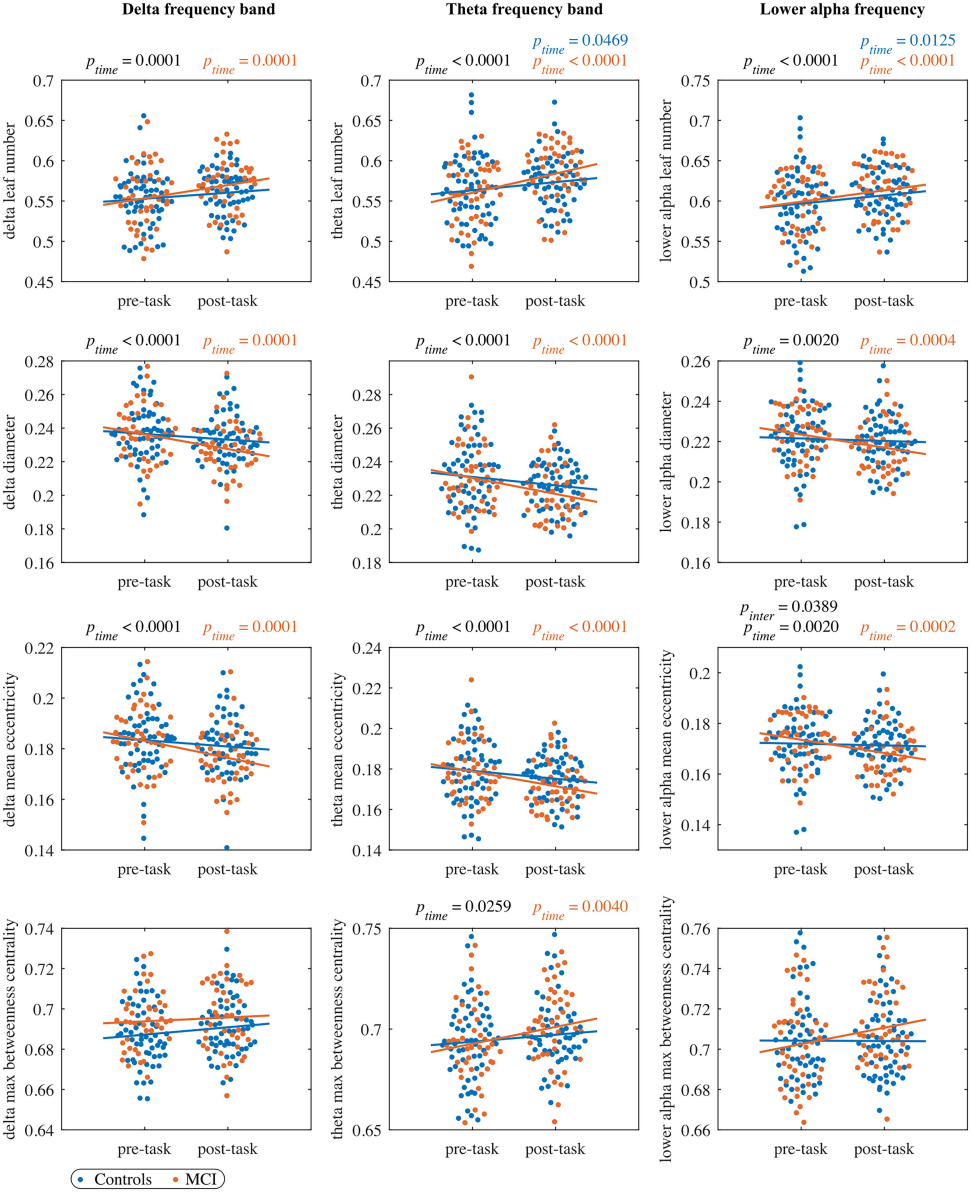
The pre- and post-task MST measures in the delta band (left column panels), theta band (middle column panels), and lower alpha band (right column panels). Each dot represents the value of one subject at pre/post-task timepoint (blue: control, orange: MCI). The black *p*_time_ and *p*_inter_ values indicate significant timepoint and interaction effects, respectively, of the LMM fitted to the whole cohort (no significant group effects were observed). The blue and orange lines represent the fit of the LMM fitted to the control and MCI group, respectively. The blue and orange *p*_time_ values indicate significant timepoint effects of the LMM fitted to the control and MCI group, respectively. All the reported *p* values are FDR-corrected.

**Figure 3:**
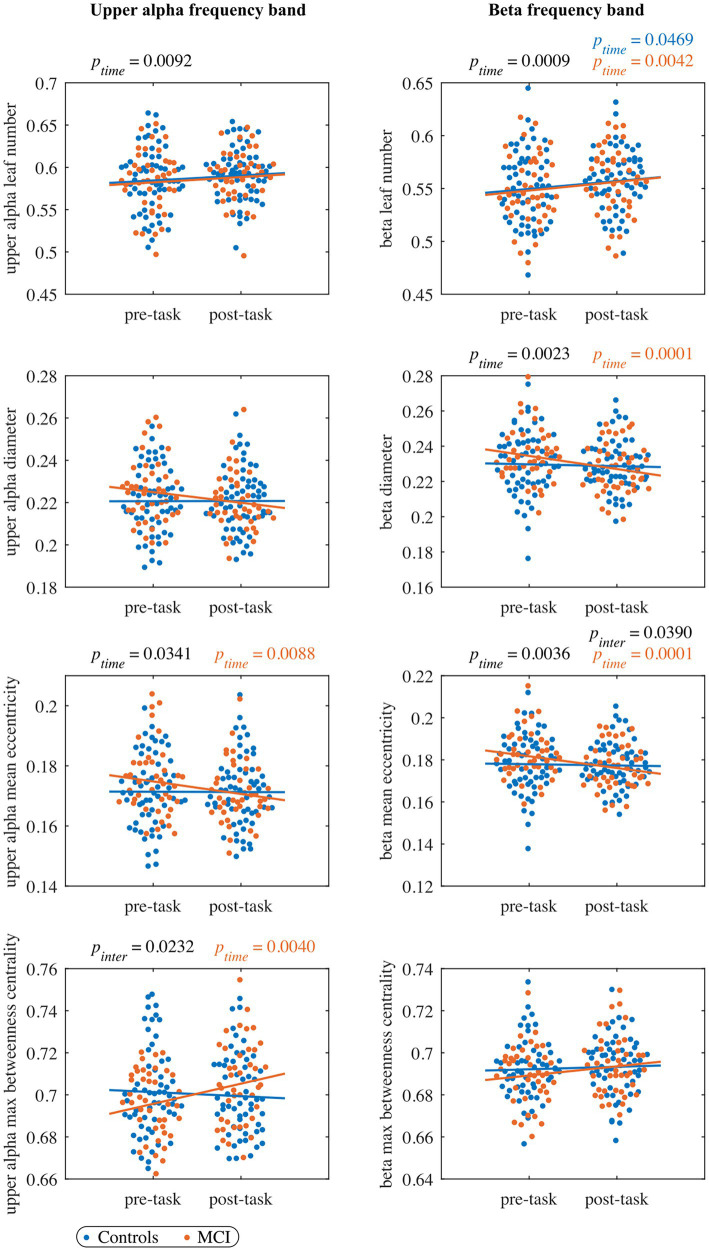
The pre- and post-task MST measures in the upper alpha band (left column panels), and beta band (right column panels). Each dot represents the value of one subject at pre-/post-task timepoint (blue: control, orange: MCI). The black *p*_time_ and *p*_inter_ values indicate significant timepoint and interaction effects, respectively, of the LMM fitted to the whole cohort (no significant group effects were observed). The blue and orange lines represent the fit of the LMM fitted to the control and MCI group, respectively. The orange *p*_time_ values indicate significant timepoint effects of the LMM fitted to the MCI group (no significant effects were observed in the control group). All the reported *p* values are FDR-corrected.

For each network measure and each frequency band, Supplementary Table 2 displays $p_{\text{FDR}}$ values for the parameters of the LMM fitted to the whole cohort, and Supplementary Table 3 gives $p_{\text{FDR}}$ values of the model adjusted to each group. Results for MST measures are also shown in Figures 2, 3, including the complete distribution of data as well as the fitting of the LMM separately for each group as a function of timepoints.

##### Delta frequency band

In the leaf number, we detected a significant timepoint effect, but we did not find a significant group or interaction effect. The leaf number significantly increased from the pre- to post-task timepoint in the MCI group, while the control group showed a trend to increase ($p_{\text{FDR}}=0.0647)$. Moreover, we observed a trend-level interaction effect in the diameter and mean eccentricity ($p_{\text{FDR}}=0.0688$ and $p_{\text{FDR}}=0.0540,$ respectively) with a significant decrease of both measures in the MCI group from the pre- to the post-task timepoint. We did not find significant timepoint, group or interaction effect in maximum betweenness centrality, global weighted clustering coefficient and weighted characteristic path length.

##### Theta frequency band

For all four MST measures, we detected a significant timepoint effect, but no significant effects of group or interaction. The leaf number and maximum betweenness centrality significantly increased from the pre- to post-task timepoint in the MCI group, while in the control group only a significant increase in leaf number was observed. Moreover, mean eccentricity and diameter demonstrated a significant decrease in the MCI group and a nonsignificant decrease in the control group from the pre- to the post-task timepoint. We did not find significant timepoint, group or interaction effect in global weighted clustering coefficient and weighted characteristic path length.

##### Lower alpha frequency band

**Figure 4:**
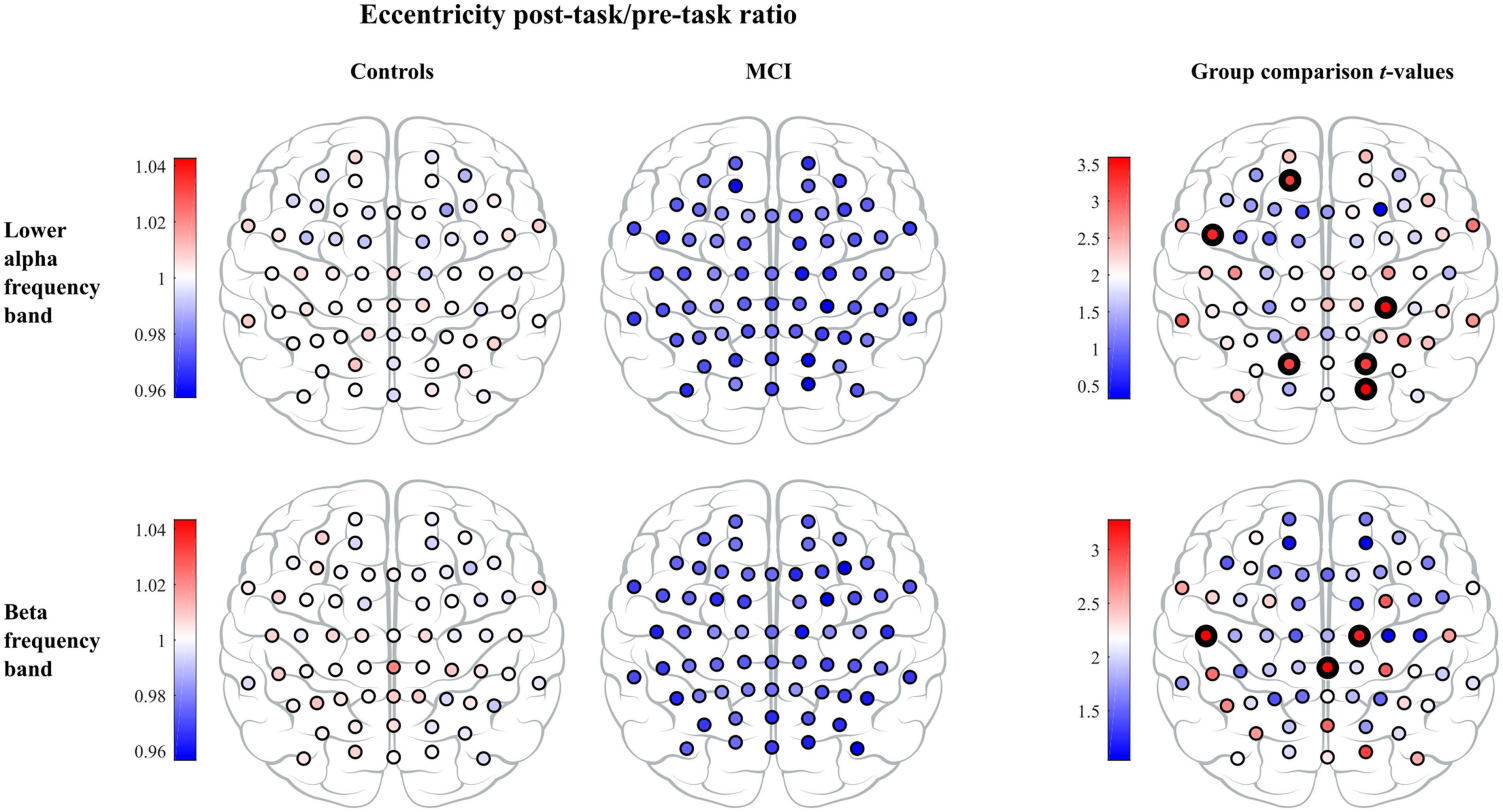
The vertex eccentricity post-task/pre-task ratio across lower alpha and beta frequency bands for controls (left column) and MCI patients (middle column). Statistics on the vertex eccentricity post-task/pre-task ratio showed significant differences between the two groups and are shown in the right column. Individual vertex *t*-values are represented with black bolded vertices indicating FWE-corrected *p*  < 0.05.

In the leaf number, we found a significant effect of timepoint, but we did not detect a significant group or interaction effect. Leaf number demonstrated a significant increase from the pre- to the post-task timepoint in both groups. Moreover, the interaction effect was significant in mean eccentricity, while a trend toward significance was observed in diameter ($p_{\text{FDR}}=0.0639$). In both cases, we observed a significant decrease in the MCI group and a nonsignificant decrease in controls from the pre- to the post-task timepoint. *Post hoc* analysis at the vertex level confirmed between-group differences in eccentricity. Compared to healthy controls, persons with MCI showed reduced vertex eccentricity post-task/pre-task ratios at all vertices with significant changes at frontal vertex AF3 ($p_{\text{FWE}}=0.0480$), central vertex CP4 ($p_{\text{FWE}}=0.0180$), left temporal vertex FT7 ($p_{\text{FWE}}=0.0327$), parietal vertices PO3 and PO4 ($p_{\text{FWE}}=0.0498$ and $p_{\text{FWE}}=0.0472$, respectively), and occipital vertex O2 ($p_{\text{FWE}}=0.0146$; see Figure 4). We did not find significant timepoint or group effect in maximum betweenness centrality. However, we observed a trend-level interaction effect ($p_{\text{FDR}}=0.0771$) in this measure with a notable increase in the MCI group from the pre- to the post-task timepoint. We did not find significant timepoint, group or interaction effect in global weighted clustering coefficient and weighted characteristic path length.

##### Upper alpha frequency band

In the leaf number, we found a significant timepoint effect, but we did not detect a significant group or interaction effect. The leaf number showed a trend of increasing from the pre- to the post-task timepoint in the control ($p_{\text{FDR}}=0.0647)$ and MCI group ($p_{\text{FDR}}=0.0550)$. Moreover, we found a trend-level interaction effect in mean eccentricity ($p_{\text{FDR}}=0.0540$) with a significant decrease in the MCI group from the pre- to the post-task timepoint. As in mean eccentricity, we observed a similar trend also in diameter. Furthermore, we detected a significant interaction effect in maximum betweenness centrality with a significant increase from the pre- to the post-task timepoint in the MCI group. We did not find significant timepoint, group or interaction effect in global weighted clustering coefficient and weighted characteristic path length.

##### Beta frequency band

In the leaf number, we observed a significant timepoint effect, but we did not detect a significant group or interaction effect. Leaf number demonstrated a significant increase in both groups from the pre- to the post-task timepoint. Moreover, the interaction effect was significant in mean eccentricity, while a trend toward being significant was observed in diameter ($p_{\text{FDR}}=0.0639$). In both cases, we detected a significant decrease in the MCI group and a nonsignificant decrease in controls from the pre- to the post-task timepoint. *Post hoc* analysis confirmed between-group differences in eccentricity. Compared to healthy controls, persons with MCI showed reduced vertex eccentricity post-task/pre-task ratios at all vertices with significant changes at central vertices C2 and CPz ($p_{\text{FWE}}=0.0430$ and $p_{\text{FWE}}=0.0304$ respectively) and left temporal vertex T7 ($p_{\text{FWE}}=0.0270$; see Figure 4). We did not find significant effects of timepoint, group or interaction in maximum betweenness centrality, global weighted clustering coefficient and weighted characteristic path length.

## Discussion

This study demonstrates that visual-based cognitive engagement has profound aftereffects on functional brain networks. Using the task aftereffect model together with MST construction we found significant changes in network organization among older adults. Importantly, the results suggest that such a model plays a significant role in distinguishing healthy controls and persons with MCI.

We have shown that network topology following the task performance does not immediately return to a resting baseline level in older adults; instead, a cognitive challenge had a significant impact on the post-task global MST topology. This impact has been seen to some extent in all five frequency bands. Importantly, although we found differences in the same direction for both groups, the task aftereffect was primarily driven by the MCI group, most notably in diameter and mean eccentricity, as well as in maximum betweenness centrality. The only measure for which a significant timepoint effect was present in both groups was leaf number. On average, the diameter and mean eccentricity decreased, while leaf number and maximum betweenness centrality increased from the pre-to the post-task timepoint, suggesting a more integrated, star-like topology in both groups. Previous studies have reported increased network integration in healthy, young adult participants during task performance. For example, the integration of fMRI functional networks initially increased during an attentional task (Breckel et al., 2013). Similarly, a higher cognitive effort led to a more globally-efficient MEG network topology during a working memory task, indicating a more integrated network architecture (Kitzbichler et al., 2011). We might speculate that a similar process that induced higher network integration during the cognitive task in former studies is also present in our case, and this might have led to the increased network integration we have observed in older adults, especially in the MCI group, from the pre- to post-task timepoint. We should note, however, that in a previous study, post-task fMRI networks in healthy, young adult participants became less integrated compared to the pre-task timepoint (Breckel et al., 2013). Our healthy controls showed negligible differences in the opposite direction. This inconsistency could be due to the age differences in two samples, use of different imaging modality, different baseline condition, different cognitive, or a combination of these factors.

We have demonstrated that reorganization in MST topology from the pre- to post-task timepoint was significantly different between MCI and control groups. Patients with MCI showed a significantly higher reduction in the mean eccentricity than healthy controls in the lower alpha and beta frequency bands. At the vertex level, we found reduced eccentricity widespread over the brain, mainly in central, left temporal, and parietal areas, but also in frontal and occipital areas, indicating that several vertices became more central in the MCI group. Additional evidence of a disruption in MCI network topology was demonstrated from a significantly higher increment in maximum betweenness centrality in the upper alpha frequency band, suggesting that more information passes through the most critical vertices in the MCI group. These results show a higher shift towards a more integrated, star-like topology from the pre- to post-task timepoint in the MCI group than in healthy controls, which breaks the balance between integration and overload prevention. Such modified topology increases global efficiency and is thus beneficial for information processing in the network, yet is less optimal as the central vertex has a greater probability to be burdened by a relatively large information flow and may require costly infrastructure (Meunier et al., 2010). A recent EEG study has reported that patients with amnestic MCI (aMCI) showed a significantly higher increment in MST hierarchy from the pre- to post-task timepoint in the beta frequency band compared to healthy controls as reflected by the percentage change from the pre- to post-task timepoint (Youssef et al., 2021). Put simply, the tree hierarchy is the ratio between the leaf number and maximum betweenness centrality and measures the balance between integration and overload prevention. We notice, however, that increased tree hierarchy does not necessarily imply a more integrated, star-like topology and needs to be interpreted with caution. For example, the interpretation depends on whether the leaf number is preserved or not; in other words, whether the leaf number influences the tree hierarchy or not. If we compare trees A and B in Figure 1, then A is more integrated than B and both the leaf number and tree hierarchy are higher in A than in B. In contrast, if we compare trees B and C in Figure 1, then B is more integrated than C, but the leaf number is the same in both trees while the tree hierarchy is smaller in B than in C. Hence, we cannot immediately conclude that increased MST hierarchy reported in Youssef et al., (2021) indicates a more integrated topology. However, in the same study one can find that the percentage of change in mean beta leaf number was not preserved from the pre- to post-task timepoint but was slightly decreased in the control group and slightly increased in the aMCI group. Consequently, it seems that together their results indicate a more integrated topology in patients, which is in line with our results. Interestingly, our and theirs did not find any significant between-group differences in the pre-task MST parameters, which supports the notion that analyzing EEG data under the task aftereffect model enhances network topology abnormalities related to MCI.

Presently, the reasons for changes in network topology between MCI patients and healthy controls are unclear, and we can only speculate which factors may contribute to this tendency.

One possible interpretation is that a more integrated network organization from the pre- to post-task timepoint in MCI patients could be a consequence of the slower recovery of MCI patients to a resting baseline level. This interpretation may be supported by a previous study, in which attentionally impaired participants showed an increased network integration in the post-task period, while more resilient subjects showed a faster recovery in the direction of the pre-task values (Breckel et al., 2013). If slower recovery lies behind the changes in network topology, this may, in turn, be related to cognitive fatigue (DeLuca et al., 2008). Alternatively, another interpretation is that the capacity of certain vertices to process incoming information might be reduced in the MCI group and that the identified patterns might be the aftereffect of greater integration of networks during task performance in MCI patients compared to controls. The reorganization of brain networks with a deviation to a more integrated, centralized topology very possibly empowers communication (recruitment) between certain specialized areas across the brain for the MCI participants which would otherwise remain unperturbed and unneeded for the controls. This integrated, centralized network organization may aid cognitive performance by increasing the flow of information through the most critical vertices among persons with MCI who seem to require more communication and recruitment across brain areas to maintain or improve task performance. Such reorganization could indicate that patients with MCI are engaged in compensatory activation, especially as both groups did not differ in their motion direction discrimination task performance (Table 1). The presence of such compensation can have important consequences. When the traffic load increases in a highly-centralized network, the most critical vertices might become congested and their neighbors start to disconnect from them. These critical vertices are therefore very sensitive to break down and can be lost with the progression of the disease (de Haan et al., 2012; Stam, 2014). The latter interpretation may be supported by past EEG studies reporting that MST network topology of the MCI group becomes more integrated during a cognitive engagement compared to healthy control. For example, such a pattern has been found while performing a visuospatial memory task in the alpha and beta frequency band (Fodor et al., 2021). Another study has shown an increased integration in MCI-AD patients under a cognitive task in the gamma frequency band (Das and Puthankattil, 2020). Future studies are needed, however, to confirm these interpretations.

To characterize network organization, we also apply a more conventional approach by computing the global weighted clustering coefficient and weighted characteristic path length of a weighted brain graph. Graphs that possess a high global clustering coefficient and a short characteristic path length exhibit healthy brain graphs—a so-called small-world organization (Watts and Strogatz, 1998; Bullmore and Sporns, 2009). Previous studies based on a single resting-state EEG recording without previous stimulation have reported a loss of small-world topology in MCI (Zeng et al., 2015; Vecchio et al., 2018; Miraglia et al., 2020). However, we found no significant group differences in the global weighted clustering coefficient and weighted characteristic path length at the pre-task timepoint. We also found no significant pre-to-post differences in these two measures over the whole cohort or between groups. This endorses the idea that MST parameters are more sensitive to detect profound changes in brain networks for MCI patients than conventional measures (López et al., 2017; Požar et al., 2020; Youssef et al., 2021).

We should note that the interpretations of our findings should be accepted with some caution. The phase lag index does not provide information about the casual or the direction of the functional connectivity edges, which is needed for a more detailed analysis of the information flow. A possible solution would be to measure effective connectivity that examines the direction of communication and estimates how likely one region influences the other. Next, we studied brain networks in sensor space. Although the phase lag index reduces the influence of volume conduction, another approach would be to conduct source space analysis.

In conclusion, our findings suggest that differences in the network topology may be promising in distinguishing healthy controls and MCI, and also in better understanding inherent differences in brain organization for MCI patients. The task aftereffect model could therefore represent a valid marker of cortical capacity and/or cortical health.

## Data availability statement

The original contributions presented in the study are included in the article/Supplementary material, further inquiries can be directed to the corresponding author.

## Ethics statement

The studies involving human participants were reviewed and approved by VK, Wayne State University, Division of Research, Institutional Review Board IRB#: 095914A. The patients/participants provided their written informed consent to participate in this study.

## Author contributions

RP, BG, and VK contributed to conception and design of the study. VK and RP provided funding. VK and BG provided supervision and project administration. TM wrote the motion stimulus software used in the intervening task. VK and KK prepared the data for analysis. RP conceptualized and wrote software for the formal analysis and visualization, and wrote the first draft of the manuscript. All authors contributed to the article and approved the submitted version.

## Funding

This research was in part supported by grants from NIA/NIH, 1R21AG046637-01A1 and 1R01AG054484, Alzheimer’s Association Award HAT-07-60437 grant, in part by the Slovenian Research Agency (research program P1-0285 and research projects J1-1694, N1-0159, J1-2451, N1-0209, J5-4423) to RP, a grant from the Slovenian Research Agency, research project P3-0366/2451 to VK and partial support for KK and BG from NIH/NIA grant P30AG053760 to the Michigan Alzheimer’s Disease Research Center.

## Conflict of interest

The authors declare that the research was conducted in the absence of any commercial or financial relationships that could be construed as a potential conflict of interest.

## Publisher’s note

All claims expressed in this article are solely those of the authors and do not necessarily represent those of their affiliated organizations, or those of the publisher, the editors and the reviewers. Any product that may be evaluated in this article, or claim that may be made by its manufacturer, is not guaranteed or endorsed by the publisher.

## Supplementary material

The Supplementary material for this article can be found online at: https://www.frontiersin.org/articles/10.3389/fnagi.2023.1061254/full#supplementary-material

## Footnotes

^1^ http://www.mingw.org

^2^The weighted distance is also referred to as weighted shortest path length in the neuroscience literature.

^3^Maximum spanning trees are more typically referred to as minimum spanning trees in the neuroscience literature. Mathematically, however, the sentence a “maximum spanning tree of a weighted brain graph is a tree that maximizes the sum of edge weights” makes more sense than the sentence “a minimum spanning tree of a weighted brain graph is a tree that maximizes the sum of edge weights.”
