# Supplementary material for: Task aftereffect reorganization of resting state functional brain networks in healthy aging and mild cognitive impairment
Source: Front Aging Neurosci. 2023 Jan 11;14:1061254. doi: 10.3389/fnagi.2022.1061254 (PMC9876535; doi:10.3389/fnagi.2022.1061254)
Supplement: Supplementary file 1 [file Table_1.DOCX]

**Supplementary Materials**

Supplementary Table 1. Resting state eyes-closed EEG global network measures for pre- and post-cognitive engagement for controls and MCI.

|  | | Pre-task timepoint | | | | Post-task timepoint | | | |
| --- | --- | --- | --- | --- | --- | --- | --- | --- | --- |
|  |  | Controls | | MCI | | Controls | | MCI | |
| **Frequency band** | **MST measure** | mean | SD | mean | SD | mean | SD | mean | SD |
| Delta | Leaf number | 0.5529 | 0.0346 | 0.5534 | 0.0382 | 0.5604 | 0.0264 | 0.5699 | 0.0335 |
|  | Diameter | 0.2365 | 0.0172 | 0.2362 | 0.0170 | 0.2332 | 0.0147 | 0.2275 | 0.0151 |
|  | Mean eccentricity | 0.1833 | 0.0131 | 0.1831 | 0.0130 | 0.1809 | 0.0112 | 0.1764 | 0.0115 |
|  | Maximum BC | 0.6872 | 0.0157 | 0.6938 | 0.0145 | 0.6909 | 0.0140 | 0.6958 | 0.0177 |
|  | Global weighted CC | 0.9636 | 0.0646 | 0.9675 | 0.0632 | 0.9615 | 0.0612 | 0.9755 | 0.0637 |
|  | Weighted characteristic PL | 0.9232 | 0.0460 | 0.9219 | 0.0410 | 0.9258 | 0.0436 | 0.9240 | 0.0459 |
| Theta | Leaf number | 0.5634 | 0.0431 | 0.5603 | 0.0401 | 0.5735 | 0.0320 | 0.5841 | 0.0365 |
|  | Diameter | 0.2309 | 0.0209 | 0.2303 | 0.0187 | 0.2259 | 0.0138 | 0.2208 | 0.0158 |
|  | Mean eccentricity | 0.1791 | 0.0160 | 0.1788 | 0.0146 | 0.1752 | 0.0108 | 0.1715 | 0.0121 |
|  | Maximum BC | 0.6937 | 0.0195 | 0.6927 | 0.0197 | 0.6972 | 0.0166 | 0.7011 | 0.0193 |
|  | Global weighted CC | 0.9239 | 0.0566 | 0.9345 | 0.0504 | 0.9228 | 0.0546 | 0.9472 | 0.0498 |
|  | Weighted characteristic PL | 1.0591 | 0.0463 | 1.0553 | 0.0320 | 1.0664 | 0.0468 | 1.0456 | 0.0444 |
| Lower alpha | Leaf number | 0.5968 | 0.0417 | 0.5993 | 0.0339 | 0.6071 | 0.0304 | 0.6133 | 0.0324 |
|  | Diameter | 0.2215 | 0.0161 | 0.2235 | 0.0130 | 0.2203 | 0.0127 | 0.2170 | 0.0126 |
|  | Mean eccentricity | 0.1720 | 0.0125 | 0.1735 | 0.0100 | 0.1713 | 0.0098 | 0.1683 | 0.0095 |
|  | Maximum BC | 0.7042 | 0.0198 | 0.7026 | 0.0220 | 0.7040 | 0.0184 | 0.7107 | 0.0192 |
|  | Global weighted CC | 1.2046 | 0.1131 | 1.2207 | 0.1166 | 1.2291 | 0.1115 | 1.2099 | 0.0974 |
|  | Weighted characteristic PL | 0.7783 | 0.0645 | 0.7769 | 0.0577 | 0.7661 | 0.0607 | 0.7784 | 0.0531 |
| Upper alpha | Leaf number | 0.5845 | 0.0378 | 0.5823 | 0.0358 | 0.5903 | 0.0313 | 0.5885 | 0.0306 |
|  | Diameter | 0.2207 | 0.0158 | 0.2249 | 0.0151 | 0.2207 | 0.0150 | 0.2199 | 0.0136 |
|  | Mean eccentricity | 0.1714 | 0.0122 | 0.1748 | 0.0116 | 0.1713 | 0.0118 | 0.1706 | 0.0101 |
|  | Maximum BC | 0.7013 | 0.0212 | 0.6958 | 0.0156 | 0.6994 | 0.0191 | 0.7053 | 0.0196 |
|  | Global weighted CC | 1.0086 | 0.0728 | 1.0032 | 0.0826 | 0.9971 | 0.0521 | 0.9949 | 0.0567 |
|  | Weighted characteristic PL | 0.9522 | 0.0558 | 0.9616 | 0.0592 | 0.9518 | 0.0543 | 0.9662 | 0.0488 |
| Beta | Leaf number | 0.5497 | 0.0350 | 0.5481 | 0.0324 | 0.5570 | 0.0284 | 0.5563 | 0.0314 |
|  | Diameter | 0.2297 | 0.0182 | 0.2345 | 0.0151 | 0.2287 | 0.0142 | 0.2270 | 0.0141 |
|  | Mean eccentricity | 0.1779 | 0.0139 | 0.1817 | 0.0112 | 0.1773 | 0.0108 | 0.1761 | 0.0106 |
|  | Maximum BC | 0.6922 | 0.0142 | 0.6892 | 0.0138 | 0.6934 | 0.0140 | 0.6936 | 0.0142 |
|  | Global weighted CC | 0.8664 | 0.0410 | 0.8714 | 0.0438 | 0.8642 | 0.0351 | 0.8680 | 0.0410 |
|  | Weighted characteristic PL | 1.2946 | 0.0307 | 1.2885 | 0.0371 | 1.2928 | 0.0311 | 1.2887 | 0.0327 |

SD, standard deviation; BC, betweenness centrality; CC, clustering coefficient; PL, path length.

Supplementary Table 2. FDR corrected $p$-values of the factors of the linear mixed model fitting each global network measure. In the $p_{FDR}$ columns * stands for statistical significance ($p_{FDR}<0.05$).

|  |  | **Timepoint** | **Group** | **Group-timepoint interaction** |
| --- | --- | --- | --- | --- |
| **Frequency band** | **MST measure** | $p_{FDR}$ | $p_{FDR}$ | $p_{FDR}$ |
| Delta | Leaf number | 0.0001* | 0.9182 | 0.2917 |
|  | Diameter | < 0.0001* | 0.7400 | 0.0688 |
|  | Mean eccentricity | < 0.0001* | 0.7360 | 0.0540 |
|  | Maximum betweenness centrality | 0.1614 | 0.1986 | 0.6700 |
|  | Global weighted CC | 0.5393 | 0.8594 | 0.4880 |
|  | Weighted characteristic PL | 0.7855 | 0.8076 | 0.9411 |
| Theta | Leaf number | < 0.0001* | 0.9182 | 0.0932 |
|  | Diameter | < 0.0001* | 0.7400 | 0.1619 |
|  | Mean eccentricity | < 0.0001* | 0.7360 | 0.1664 |
|  | Maximum betweenness centrality | 0.0259* | 0.9626 | 0.4004 |
|  | Global weighted CC | 0.5393 | 0.6080 | 0.4600 |
|  | Weighted characteristic PL | 0.7855 | 0.6823 | 0.2884 |
| Lower alpha | Leaf number | < 0.0001* | 0.9182 | 0.6876 |
|  | Diameter | 0.0020* | 0.9838 | 0.0639 |
|  | Mean eccentricity | 0.0020* | 0.9286 | 0.0390* |
|  | Maximum betweenness centrality | 0.0982 | 0.9626 | 0.0771 |
|  | Global weighted CC | 0.5393 | 0.9146 | 0.2537 |
|  | Weighted characteristic PL | 0.7855 | 0.6823 | 0.3042 |
| Upper alpha | Leaf number | 0.0092* | 0.9182 | 0.9195 |
|  | Diameter | 0.0620 | 0.7400 | 0.0688 |
|  | Mean eccentricity | 0.0341* | 0.7360 | 0.0540 |
|  | Maximum betweenness centrality | 0.0982 | 0.9964 | 0.0232* |
|  | Global weighted CC | 0.3305 | 0.9146 | 0.8497 |
|  | Weighted characteristic PL | 0.7855 | 0.6823 | 0.9080 |
| Beta | Leaf number | 0.0009* | 0.9182 | 0.9195 |
|  | Diameter | 0.0023* | 0.7400 | 0.0639 |
|  | Mean eccentricity | 0.0036* | 0.7360 | 0.0390* |
|  | Maximum betweenness centrality | 0.1230 | 0.9626 | 0.4286 |
|  | Global weighted CC | 0.5393 | 0.8594 | 0.8497 |
|  | Weighted characteristic PL | 0.7855 | 0.6823 | 0.9080 |

CC, clustering coefficient; PL, path length.

Supplementary Table 3. Effect of timepoint on global network measures in each of the groups. FDR corrected $p$-values of the linear mixed model fitting each global tree measure in controls and MCI independently. In the $p_{FDR}$ columns * stands for statistical significance ($p_{FDR}<0.05$). Group models were evaluated only in the case of significant effect of timepoint or the interaction between timepoint and group in the linear mixed model fitted to the whole cohort.

|  |  | **Controls** | **MCI** |
| --- | --- | --- | --- |
| **Frequency band** | **MST measure** | $p_{FDR}$ | $p_{FDR}$ |
| Delta | Leaf number | 0.0647 | 0.0001* |
|  | Diameter | 0.1236 | 0.0001* |
|  | Mean eccentricity | 0.1814 | 0.0001* |
| Theta | Leaf number | 0.0469* | < 0.0001* |
|  | Diameter | 0.1236 | < 0.0001* |
|  | Mean eccentricity | 0.1565 | < 0.0001* |
|  | Maximum betweenness centrality | 0.4452 | 0.0040* |
| Lower alpha | Leaf number | 0.0125* | < 0.0001* |
|  | Diameter | 0.5631 | 0.0004* |
|  | Mean eccentricity | 0.8530 | 0.0002* |
| Upper alpha | Leaf number | 0.0647 | 0.0550 |
|  | Mean eccentricity | 0.9388 | 0.0088* |
|  | Maximum betweenness centrality | 0.4452 | 0.0040* |
| Beta | Leaf number | 0.0469* | 0.0042* |
|  | Diameter | 0.5834 | 0.0001* |
|  | Mean eccentricity | 0.8530 | 0.0001* |
